# Assessing the Feasibility of a Peer Education Project to Improve Mental Health Literacy in Adolescents in the UK

**DOI:** 10.1007/s10597-022-01059-w

**Published:** 2023-01-16

**Authors:** Abigail Emma Russell, Esther Curtin, Emily Widnall, Steven Dodd, Mark Limmer, Ruth Simmonds, Judi Kidger

**Affiliations:** 1 Children and Young People’s Mental Health Research Collaboration, University of Exeter Medical School, University of Exeter, Exeter, UK; 2 Centre On Climate Change and Planetary Health, London School of Hygiene and Tropical Medicine, London, UK; 3 Population Health Sciences, Bristol Medical School, University of Bristol, Bristol, UK; 4 Faculty of Health and Medicine, Lancaster University, Lancaster, UK; 5 Mental Health Foundation, London, UK

**Keywords:** Peer education, Mental health literacy, School-based intervention, Feasibility

## Abstract

Many mental health problems begin in adolescence and occur on a spectrum of severity: early recognition and intervention is important. This study is a quantitative feasibility study of the Mental Health Foundation’s Peer Education Project (PEP). Attrition, psychometric properties of questionnaires, indications of improvement on a range of outcomes, and sample size required for a powered trial of effectiveness were assessed. 203 students completed the survey both pre and post-intervention. It was found that existing previously-validated measures had good psychometric properties, with two new questionnaires demonstrating reasonable reliability (self-help confidence alpha = 0.78, mental health knowledge alpha = 0.59). There were indications of improvement in help-seeking intentions, the number of sources likely to seek help from, and mental health knowledge from pre- to post-intervention. A future trial of PEP with a sample of approximately 36 schools, researcher-led data collections, and help-seeking intentions or sources as a primary outcome appears to be feasible.

**Supplementary Information:**

The online version contains supplementary material available at 10.1007/s10597-022-01059-w.

## Introduction

Mental health problems in young people are prevalent in the UK, with one in six young people from the general population of England meeting criteria for a psychiatric disorder in 2017 (NHS Digital, 2021), one in four young people reporting self-harm (Kidger et al., 2012; Patalay & Fitzsimons, 2021), and the last decade has seen increasing rates of emotional disorders in teenage girls (NHS Digital, 2018). Poor mental health during childhood and adolescence negatively impacts on educational and socio-emotional outcomes for young people (Sallis et al., 2019), and also predicts poor life course trajectories with further disorders more likely to occur. The knock-on effects of impaired functioning often lead to poor occupational, economic and relational outcomes (Aebi et al., 2014; Asselmann et al. 2018; Copeland et al., 2015). Evidence-based approaches for prevention and intervention to improve young people’s mental health are therefore necessary. Current mental-healthcare systems in the UK are in high demand, with long waiting lists and high ‘thresholds’ of symptoms and impairments necessary to access professional support (Children’s Commissioner for England, 2020). However, because many mental health problems occur on a spectrum of severity, early recognition and intervention gives opportunities for preventing the deterioration of symptoms, building coping strategies to manage problems that are emerging or less severe, and to identify how, when and what professional or community support to seek (Colizzi et al., 2020). To reach the widest number of young people, universal prevention programmes set in schools are increasingly being explored as avenues for intervention delivery (O’Reilly et al., 2018; Weare, 2013).

For a preventative intervention to be implementable and sustainable in the UK school context, considerations around mode of delivery, training and cost are critical (Langley et al., 2010). One widely-used but poorly evaluated behaviour change method that shows promise is peer-delivered health education (henceforth referred to as peer education) (Harden et al., 2016; Mason, 2003). This is when young people themselves are trained to deliver an intervention to their peers (Shiner, 1999). The evidence base for peer education remains weak, with most studies assessing these interventions focusing on physical health promotion, such as sexual and reproductive health, HIV awareness or dental hygiene (Harden et al. 1999). There are few studies of peer education for mental health prevention/promotion (King & Fazel, 2021). Many existing studies using the peer education approach are conducted in low and middle-income countries, where professional health service access and training are limited, and the potentially wide-reaching impacts of training a small number of students to deliver an intervention to a large number of peers at low cost is of importance. There are varied models hypothesising how peer education would generate impact and improve outcomes based on the principles and theories of behaviour change (e.g. Bandura & Walters, 1977): key elements identified include adolescents considering other young people to be accurate and reliable sources of information and being more likely to seek support from their peers, existing social networks being leveraged and extended, and peer educators modelling good practice and behavior (Johnson et al., 2015; Rickwood et al., 2005).

Peer education provides the basis of the Mental Health Foundation's UK-wide programme, that aims to decrease stigma and improve knowledge and literacy around mental health in secondary school aged young people (11–18 years old). The ‘Peer Education Project’ (PEP) is currently available for secondary schools to purchase, and entails online training for staff, who then select and train ‘Peer Educators’ (typically Year 12 students age 16–17 years). Peer Educators deliver a series of five classroom lessons around mental health to younger students (usually Year 7, age 11–12 years) to meet the aims of the project. The lessons are interactive and cover mental health awareness, risk and protective factors, ways to ‘stay well’, the importance of seeking help and support for mental health, and supporting others with their mental health. It has been evaluated on a small-scale previously, showing improved knowledge and confidence in discussing mental health. However this evaluation had limitations (Eisenstein et al. 2019).

We have conducted a feasibility study and realist evaluation, with the view to conducting a full-scale effectiveness study, and in this article we report the findings from this feasibility study. A previous publication outlines the protocol for this evaluation. We report on four research questions: (1) What is the likely attrition from the intervention and the study?, (2) What are the psychometric properties of the measures used to assess the PEP?, (3) Is there an indication that the PEP can improve mental health literacy, as assessed by self-help and help-seeking intentions and confidence, peer support, and mental health knowledge and wellbeing; are there specific user groups that benefit more from the PEP than others?, and (4) What sample size is required for a full trial?

## Methods

### Study Design and Sample

Mainstream secondary schools in two geographical areas of England; North and the South West, were approached to participate in this feasibility study. The intention was to recruit schools prospectively to the intervention to start the PEP either during the 2020/2021 or 2021/22 academic year, however due to the multiple COVID-19 lockdowns and associated challenges for schools to engage in additional activities, recruitment was broadened to include schools already signed-up to the PEP through the Mental Health Foundation. We took a joint approach to recruitment, which entailed sending out an initial email from the research team and our partner from the Mental Health Foundation. From the 47 schools approached, three were recruited from the initial contact, two from a later contact, and two were contacted directly by the Mental Health Foundation as they had already signed up. Six schools participated in the intervention arm and one additional school (a grammar school) was recruited as a validation school to assess test–retest reliability of novel PEP-specific measures designed as part of this study (did not receive the intervention). Headteachers provided informed consent for their schools’ participation and signed research agreements prior to data collection commencing. Peer Educators and Peer Learners were recruited by each school through staff selecting appropriate older students to act as Peer Educators, and selecting a Peer Learner year group. Although the intervention was originally designed for year 12 (age 16–17 years) and year 7 (age 11–12 years) students, respectively, some schools chose to use different year groups. The intervention content was originally designed to meet the literacy age of Year 7’s, and was tailored to students new to the secondary school setting with a relatively basic level of knowledge about mental health. Year 12 were selected to deliver the intervention as they were felt to be suitably mature to handle the content and demands of delivering lessons to their peers, and this year group have more time available in their timetables and less exam pressures than the year groups on either side of them (year 13 and 11). Parents were sent study information sheets with the option to opt their child out of the study (no parents did this). Thereafter, teachers sent the study team the students’ school email addresses in a password protected spreadsheet, which totaled 207 peer educators and 1209 peer learners across the six intervention schools, and 190 students of the peer learner age group in the validation school.

### Measures

Prior to the PEP being delivered, students completed an online survey (via REDCap, see (https://projectredcap.org/)), comprising six questionnaires and questions about sociodemographic information. The study team agreed on a set date and time to send the survey to students’ email addresses according to when teachers said they could dedicate supervised lesson time (around 25 min); however only three schools confirmed lesson time was allocated. Students completed the same survey at follow-up, ranging from one week to three months after the intervention completed (one school finished the intervention just before the summer holiday, resulting in the shorter follow up period). All students provided informed consent before commencing each survey.

The following measures were completed for both peer educators and peer learners:

*Help-seeking intentions using an adapted version of the General Help-Seeking Questionnaire (GHSQ) *(Olivari & Guzmán-González, 2017). This 11-item questionnaire measures intentions to seek help from a variety of sources for personal/emotional problems, with students asked to rate on a seven-point Likert scale their likelihood of approaching each source for help from “extremely unlikely” to “extremely likely”. The sources included a range of formal and informal contacts, adapted to cover varied contacts inside and outside of school. Data were utilised in two ways: First, scores were summed and then averaged, giving a total score from 1 to 7, with higher scores indicating greater help-seeking intentions (henceforth referred to as *‘help-seeking intentions*’). Secondly, the number of help sources scored from likely to extremely likely (scores from 5 to 7) was derived, based on the assumption that having less sources whom you are *more likely* to seek help from may be better than having more sources whom you are *less likely* to seek help from; henceforth *‘number of sources likely to see help from’*.

*Self-help confidence* This newly-developed measure (piloted and revised with student feedback) assessed reported confidence in supporting mental health. The 12 items were purpose-developed with stakeholder engagement and consultation; three items asked about confidence in talking about their own mental health, three items about knowing where to find information about mental health, three items relating to coping with feelings and taking care of mental health, and three items around confidence in supporting a friend whose mental health a student is concerned about. For each item, students rated their agreement on a four-point Likert scale from ‘strongly disagree’ to ‘strongly agree’, with higher scores indicating greater help-seeking confidence (range 12–48).

*Mental health knowledge* relevant to the intervention content, was assessed by 12 items, e.g., ‘mental health is something we all have’ and ‘people with mental health problems can get better’. This measure was also purpose-developed for this study as above. Students chose from four response options from ‘completely untrue’ to ‘completely true’ and scores were summed with higher score indicating greater mental health literacy (range 12–48).

*Perceived peer support* was assessed using a subscale of the Sense of Belonging Scale (Hoffman et al. 2002). This eight-item scale is one of five subscales included in the original instrument. There are five response options from ‘completely untrue’ to ‘completely true’, higher score means greater peer support (range 8–40).

*Mental wellbeing* was measured using the 7-item short version of the Warwick–Edinburgh Mental Wellbeing Scale (SWEMWBS) (Melendez-Torres et al. 2019), that measures subjective and psychological well-being, and has shown easy administration and validity among adolescents as well as being responsive to change. Students rated how they had been feeling over the past two weeks by selecting one of five response options from 'none of the time’ to ‘all of the time’. Higher scores indicate better wellbeing (range 7–35).

Sociodemographic information was also captured: the *Family Affluence Scale* (FAS) (Boyce et al., 2006) consists of six items which are summed together into an aggregated FAS index ranging from 0 to 13. This is commonly used as a proxy for socioeconomic status (SES) in studies of children and young people, with good external criterion validity with wealth indicators. Demographics self-reported by young people included their school, whether they were a peer learner or peer educator, year group, gender, FSM status, sexual orientation, ethnicity and presence of a disability. Participants were asked ‘which of the following best describes your gender’ and were given four possible responses: ‘male’, ‘female’, ‘prefer to self-describe' and ‘prefer not to say’. The self-described responses were recoded to make the following categories: ‘gender fluid’, ‘non-binary' and ‘other/unknown’, and were further combined into ‘other’ for the regression analyses. Similarly, disability was ascertained by the item ‘are your day-to-day activities limited because of a health problem or disability which has lasted, or is expected to last, at least 12 months?’ with the responses combined to create a binary ‘yes’/’no’ variable for the analyses.

### Analysis

We first examined the characteristics of schools and students that participated in the validation and intervention arms, reporting information on the number of students recruited, consented and responding to the baseline and follow-up questionnaires. See Figure S1 for a flowchart of participation and attrition throughout the study. We calculated demographic and baseline summary statistics for the six schools that received the intervention (see Table 1), and assessed whether the validation school students were markedly different using t-tests and chi^2^ statistics. All analyses were conducted in Stata v17/18 (StataCorp). Table S1 reports descriptive information on the number of valid responses to questionnaires and the number of missing data. Listwise deletion (removing any participant with missing data on any measure from all analyses) was considered inappropriate due to the low number with complete responses across all questionnaires. Information on treatment of missing data and generation of an imputed dataset are included in the Supplementary Material.

**Table 1:** School-level characteristics and response rates for the intervention schools (n = 6) and validation school (n = 1)

Area	% FSM eligible	Peer educator year group	Peer learner year group	Response rate baseline N/n eligible (%)	Response rate follow-up N/n eligible (%)	Length of gap post-intervention
Southwest	NA	12	7	86/109 (79.0)	45/109 (41.3)	12 weeks
Southwest	14.2%	13	8, 9	153/549 (27.9)	172/549 (31.3)	1 week
Southwest^a^	NA	12	7, 8	95/126 (75.4)	9/126 (7.14)	3 weeks
Northern	49.4%^c^	12, 13	7	56/81 (69.1)	51/81 (63.0)	5 weeks
Northern	12.6%	13	7	142/349 (40.7)	0/349 (0.00)	NA
Northern	26.7%	10	7	63/202 (31.2)	139/202 (68.8)	12 weeks
				Total: 595/1416 (42.0)^d^ Mean per cluster: 365.3/6 (60.9)^f^	Total: 416/1416 (29.4)^e^ Mean per cluster: 240.1/6 (40.0)	Average: 6.6 weeks
Northern ^b^	4.9%	NA	NA	175/190 (92.1)	138/190 (72.6)	

*FSM* free school meal, *N* eligible defined as total number of email addresses provided by the school

^a^Existing user school

^b^Validation school

^c^Above national average (27.7%)

^d^597 students consented but 2 school IDs are missing

^e^597 students consented but 2 school IDs are missing

^f^Unit of cluster = school

The analysis of psychometric properties of the measures at baseline was conducted using data from those who had complete data on each measure at baseline and follow-up (the original dataset, sample sizes vary by measure) and the imputed dataset separately. We assessed the psychometric properties of the outcome measures, using baseline data from all schools (including the validation school) to assess validity and reliability, and using data just from the validation school to assess test–retest reliability. Reliability was assessed by calculating Cronbach's alpha. Test–retest reliability was assessed by calculating the intraclass correlation coefficient (ICC). The ICCs were calculated using a two-way mixed effects model. The *kappaetc, icc(mixed, blend)* command was used in Stata v17.0 to generate ICCs and 95% confidence intervals. To assess validity of the new questionnaire measures, exploratory and confirmatory factor analysis were conducted using an iterated principal factors approach with oblique rotation for the mental health knowledge scale and the help-seeking confidence scales, using the *factor* command in Stata v17. Further information on the factor analysis methods is outlined in the Supplementary material.

To assess whether there was an indication that the PEP can improve mental health literacy, using the imputed dataset, we conducted paired samples t-tests to assess change in each of the six outcome variables (average help-seeking intentions, number of sources likely to seek help from, self-help confidence, mental health knowledge, peer support, and mental well-being) from baseline to follow-up for individuals with data at both time-points. To assess whether there were specific user groups who benefitted more than others, we conducted a series of multiple linear regression models, for each of the five outcome variables, controlling for the baseline scores and school. Gender, student type (peer educator, peer learner), fidelity in peer educator age group (i.e., using the recommended year group of year 12), and fidelity in peer learner group age (i.e., using the originally recommended year group of year 7) were the predictors assessed. The larger groups served as the reference categories, so that a positive coefficient meant that the outcome was higher for those in the other categories. Visual inspection of the Q-Q plots of the residuals of each model was used to ensure any deviations from normality did not affect the results. Given that this study was not designed to be powered to evaluate effectiveness, these findings should be considered exploratory and tentative.

Finally, to assess sample size needed for an appropriately-powered trial, decisions were made following discussion about the most proximal changes that would be expected to be seen if the intervention was achieving its aims. The potential future primary outcomes were mental-health knowledge, help-seeking intentions and self-help confidence. For each, we utilised the intra-cluster correlation coefficient and 95% CIs for each scale at baseline, the mean cluster size assuming 30% attrition of all students approached (and a mean of 170 eligible peer learners per school), an effect size of 0.3 for each measure and the standard deviation from the baseline sample. This information was used to determine the sample size (N schools) required to achieve more than 90% power at an alpha level of 5% if the intra-cluster correlation coefficient were to be the reported value at baseline. In addition, we examined intra-cluster correlation coefficients from other similar studies and measures and calculated sample sizes for a coefficient of 0.05 based on these previous ICCs (Parker et al., 2021).

## Results

### School and Student Participation

Six schools participated in the intervention arm (one of which had previously implemented PEP with other year groups, as the intervention has been available to subscribing schools for several years), comprising two fee-paying single-sex girls’ schools and four state mixed schools. Of the six schools, three of the schools delivered the intervention to the target age (year 7, range year 7-year 9: age 11–14 years), with three delivering the intervention to year 8 or 9 Two of the six schools used the target age for the peer educators (year 12, with the other four using students in year 10-year 13 as peer educators: age 14–18 years). All schools completed baseline measures before the intervention. The follow-up survey was completed one to 12 weeks post-intervention. One school did not engage in the follow-up survey. One additional school was recruited as a validation school (a boys grammar school) to assess test–retest reliability of the new measures, and completed baseline and follow-up exactly four weeks apart. Three intervention schools dedicated class time for survey completion at baseline and two schools did so at follow-up, and one school allowed students to complete during class or in their own time, with the other schools requiring students to complete the survey in their own time. The validation school dedicated class time at both time-points. Table 1 shows the school-level response rates and characteristics. Five-hundred and ninety-seven students participated in the baseline questionnaire from the intervention schools and 417 participated at follow-up. Of these, 203 participated at both time-points, however due to missing data, the number of complete responses varied across measures, ranging from 159 for peer support to 185 for help-seeking intentions. Certain students either only had data at baseline (n = 394) or follow-up (n = 214). It should be noted that the unique circumstances of the Covid-19 pandemic resulted in substantial student absence during the data collection periods. In the validation school, 175 students participated at baseline, 137 at both time-points, and one only at follow-up, and it was therefore not possible to ascertain what the likely attrition from the study or intervention would be in usual circumstances, beyond reports from all schools that they delivered PEP. See Figure S1 for a flowchart of participation through the study.

Descriptive statistics at baseline for the intervention and validation schools were reported separately, and the results showed a number of differences between the groups (see Table 2). Examination of the distribution of scores indicated that all questionnaires were broadly normally distributed, with the exception of the peer support scale which was slightly negatively skewed.

**Table 2:** Descriptive statistics for demographic and outcome variables at baseline for the intervention (n = 6) and validation school (n = 1) separately with associated sample size indicated

Variables (demographic)	Intervention schools		Validation school		*p*-value
	*n*	*n* (%)	*n*	*n* (%)	
School year	590		175		** < 0.0001**
7		259 (43.9)		175 (100.0)	
8		100 (17.0)			
9		70 (11.9)			
10		19 (3.22)			
12		139 (23.6)			
13		3 (0.51)			
Student status	586		NA		
Peer educator		161 (27.4)			
Peer learner		425 (72.5)			
Gender	516		166		** < 0.0001**
Male		157 (30.4)		162 (97.6)	
Female		331 (64.2)			
Gender fluid		3 (0.58)			
Non-binary		5 (0.97)			
Other, unknown		3 (0.58)			
Prefer not to say		17 (3.29)		4 (2.41)	
FSM eligible	517		166		**0.017**
Yes		43 (8.32)		7 (4.22)	
No		439 (84.9)		155 (93.4)	
Not sure		35 (6.77)		4 (2.41)	
Sexuality	514		166		** < 0.0001**
Straight		370 (72.0)		126 (75.9)	
Gay/lesbian		20 (3.89)			
Bisexual/pansexual		45 (8.75)		1 (0.60)	
Asexual/aromantic		8 (1.56)		2 (1.20)	
Not sure		42 (8.17)		25 (15.1)	
Prefer not to say		29 (5.64)		12 (7.23)	
Ethnicity	516		165		** < 0.0001**
White		415 (80.4)		70 (42.4)	
Mixed		35 (6.78)		8 (4.85)	
Asian/Asian British		21 (4.07)		71 (43.0)	
Black/African/Caribbean/Black British		27 (5.23)		11 (6.67)	
Chinese		9 (1.74)			
Arab		2 (0.39)		2 (1.21)	
Other		7 (1.36)		3 (1.82)	
Disability	513		165		0.15
Yes		61 (11.9)		13 (7.88)	
No		452 (88.1)		152 (92.1)	

	*n*	Mean (SD)	*n*	Mean (SD)	
FAS score	508	9.38 (2.25)	162	9.88 (1.92)	**0.011**

Variables (outcomes)	*n*	Mean (SD)	Min, max	*n*	Mean (SD)	Min, max	*p*-value
Mean help-seeking intentions^a^	579	3.42 (1.06)	1, 6	175	3.80 (0.96)	1.45, 6.36	** < 0.0001**
Friend	558	5.11 (1.53)	1, 7	172	4.53 (1.41)	2, 7	** < 0.0001**
Parent	565	5.20 (1.78)	1, 7	172	6.14 (1.15)	1, 7	** < 0.0001**
Other relative/family member	561	3.91 (1.76)	1, 7	168	4.64 (1.44)	1, 7	** < 0.0001**
Teacher	556	3.19 (1.60)	1, 7	172	4.09 (1.56)	1, 7	** < 0.0001**
Other adult in my school	562	2.80 (1.58)	1, 7	170	3.23 (1.43)	1, 7	**0.001**
An older student in my school	566	2.40 (1.43)	1, 7	174	2.55 (1.43)	1, 7	0.24
Adult outside of my school	556	1.69 (1.70)	1, 7	174	3.03 (1.46)	1, 7	**0.020**
Mental health professional	560	3.83 (1.88)	1, 7	171	4.0 (1.84)	1, 7	0.38
Doctor/GP	559	3.25 (1.72)	1, 7	174	3.73 (1.65)	1, 7	**0.001**
Phone helpline	563	2.69 (1.76)	1, 7	169	3.08 (1.83)	1, 7	**0.010**
Online support	562	2.40 (1.58)	1, 7	174	2.76 (1.57)	1, 7	**0.0090**
Number of sources likely to seek help from^b^	579	2.57 (2.10)	1, 7	175	3.43 (2.22)	0, 11	**0.0001**
Self help confidence^c^	558	34.8 (5.00)	16.4, 47	172	36.5 (4.56)	14, 48	**0.0001**
Mental health knowledge^d^	534	39.1 (3.70)	24, 48	175	38.4 (3.03)	31, 49.2	**0.020**
Peer support^e^	522	29.6 (6.20)	11, 40	172	29.5 (5.54)	9, 40	0.86
Mental well-being^f^	519	22.3 (5.36)	7, 35	171	24.8 (4.45)	7, 35	**0.0001**

Bold values indicate *p* < 0.05

### What are the Psychometric Properties of the Measures Used to Assess the PEP: Internal Consistency

Across all participants, Cronbach’s alpha for complete questionnaires ranged from 0.59 to 0.84 (Table 3). The established measures (General Help-seeking Questionnaire, Sense of Belonging scale and SWEMWBS) all had alphas of > 0.8, indicating excellent reliability. Of the two new questionnaires being assessed, the self-help confidence measure had good reliability (alpha = 0.78), however the mental health knowledge scale had a lower reliability of 0.59. After assessing correlations between the items on this measure, we removed two and re-calculated the alpha, however this resulted in almost no change (alpha = 0.60). Calculating the alphas separately for peer educators and peer learners showed greater reliability across general help-seeking and help-seeking confidence for the peer learners at baseline, with peer educators’ responses to the mental health knowledge scale being more consistent than those of younger students. Supplementary Table S2 shows inter-item covariance averages, and alphas for all scales as well as for additional groupings within questionnaires (matching with the hypothesised factors in the confirmatory factor analysis (CFA)).

**Table 3:** Reliability of questionnaires (Cronbach’s alpha at baseline and ICC)

Scale	All participants, baseline			Validation school only, one-month test–retest (*N* = 169)			
	Cronbach's alpha			Intraclass correlation coefficient	95% CI (lower)	95% CI (upper)	*p*
	All	Peer learners	Peer educators				
General help seeking	0.84	0.85	0.80	0.69	0.69	0.82	< 0.001
Self-help confidence statements	0.78	0.80	0.72	0.67	0.66	0.80	< 0.001
Mental health knowledge	0.59	0.55	0.65	0.50	0.47	0.68	< 0.001
*Mental health knowledge*^a^	*0.60*	*0.54*	*0.68*				
Sense of belonging: peer relationships	0.80	0.78	0.80	0.79	0.76	0.86	< 0.001
SWEMWBS	0.85	0.84	0.84	0.73	0.69	0.82	< 0.001

^a^10 items removing two with low correlations; *SWEMWBS* Short Warwick-Edinburgh Mental Wellbeing Scale

### Test–Retest Reliability

ICC estimates and 95% confidence intervals were calculated for all the questionnaires at baseline in the validation school sample only (N = 169, all peer learner age; see Table 3), and repeated in the imputed dataset (Supplementary Table S3). Coefficients of test–retest reliability were generally high and satisfactory for established questionnaires (e.g. 0.69, 95% CI 0.69, 0.82 for the general help-seeking questionnaire, 0.79 (95% CI 0.76, 0.86) for the sense of belonging scale). The ICC for the self-help confidence scale was 0.67 (95% CI 0.66, 0.80), and for the mental health knowledge questionnaire was 0.50 (95% CI 0.47, 0.68).

### Exploratory Factor Analysis

Sampling adequacy was adequate at between 0.72 and 0.82 for the three scales that we included in the factor analysis (help-seeking intentions, self-help confidence and mental health knowledge), and p-values for the test of sphericity were all < 0.001, indicating that correlations between items were sufficiently large for factor analysis. For self help confidence, exploratory factor analysis (EFA) indicated that a three-factor solution was the best fit for the data, determined by Eigenvalues of > 0.8. Following oblique rotation, seven items were in one factor (would tell a friend to speak to an adult if worried about their mental health, feel OK talking about my mental health with other people, knowing how to explain to someone about how I’m feeling and talking to someone about mental health in spite of how they might react, knowing how to take care of my mental health, knowing when to ask for help with how I am feeling and knowing how to use a breathing exercise to manage how I am feeling); three items loaded onto a second factor (knowing where to get information in school to look after my mental health, knowing who I can talk to if I want to know more about mental health, and knowing where to get help and support for my mental health in school); and two items loaded onto a third factor (if I was worried about a friends mental health I; would talk to a friend about their mental health, and would [not] be too embarrassed to do anything about it). Factor loadings for each item are shown in Supplementary Table S4 along with the questions within each factor. EFA of the mental health knowledge questionnaire indicated that a two-factor solution was the best fit to the data based on Eigenvalues of > 0.7. The first factor included: mental health is something we all have, people with mental health problems can get better, physical activity (exercise) can improve mental health, there’s not much you can do to help a friend with a mental health problem, the amount of sleep people get can affect how they feel, and what people eat and drink can affect their mental health. The second factor included: having good mental health means there are no problems in your life, very few people experience mental health problems, problems with friends or classmates can make your mental health worse, the environment people live and grow up in can affect our mental health, noticing or paying attention to negative emotions can make them worse, and people who have mental health problems can find it difficult to do school work. Factor loadings are shown in Supplementary Table S5 along with the questions in each factor.

### Confirmatory Factor Analysis

Model fit statistics for the confirmatory factor analysis of the suggested 3-factor solution for the self help confidence statements considered by the authors and the 3-factor solution that emerged from the EFA were examined. The model generated by the EFA was a better fit to the data, with a root mean square error of approximation (RMSEA) of 0.05 (95% CI 0.05, 0.06), however the model had a X^2^(51) of 160.58, p < 0.001, indicating the suggested model was not a good fit in comparison to the saturated model (Xia and Yang 2019). The model fit statistics for the author-suggested model were poor overall, with a comparative fit index (CFI) 0.75, Tucker-Lewis index (TLI) 0.68 and a significant X^2^ compared with the baseline model (see Table 4) (Hu, 1999, 2009). Table S6 shows the factor loadings for each item and the R^2^ values for all CFA models. The two models tested for the general help-seeking questionnaire also showed poor overall model fit (3-factor X^2^(41) = 655.38, p < 0.001; 4-factor X^2^(38) = 408.29, p < 0.001). The CFI and TLI values were below 0.9 for both models, although the 4-factor solution had a better fit than the three factor (CFI 3-factor 0.79, 4-factor 0.87; Table 4). The RMSEA values and their confidence intervals were also statistically significant at p < 0.001, overall indicating a poor model fit. Factor loadings and R^2^ for each model are presented in Table S6. The likelihood ratio test did however support that the 4-factor model was a significantly better fit than the 3-factor (p < 0.001).

**Table 4:** Confirmatory factor analysis model fit statistics

Model	Help-seeking confidence 3-factor solution (author suggestion)	Help-seeking confidence 3-factor (suggested by EFA)	GHS 3 factor solution	GHS 4 factor solution
Measure of fit				
X^2^ (compared with saturated model)	570.176	160.583	655.376	408.29
Degrees of freedom	51	51	41	38
*p*-value	< 0.001	< 0.001	< 0.001	< 0.001
AIC	18,143.798	17,734.205	28,720.84	28,479.755
BIC	18,323.51	17,913.917	28,887.402	28,660.197
CFI	0.752	0.948	0.786	0.871
TLI	0.679	0.932	0.713	0.814
RMSEA (95% CI)	0.12 (0.11, 0.13)	0.05 (0.05, 0.06)	0.14 (0.13, 0.15)	0.11 (0.10, 0.12)
p-value (probability RMSEA < = 0.05)	< 0.001	0.236	< 0.001	< 0.001

*AIC* Akaike information criterion, *BIC* Bayesian information criterion, *CFI* comparative fit index, *TLI* Tucker–Lewis Index; *RMSEA* root mean square error of approximation

### Change in Scores from Baseline to Follow-Up

Table 5 shows the outcome variables at baseline and follow-up for the participants with data from both time-points. At follow-up, after adjusting for baseline, mean help-seeking intentions (n = 185, mean difference = 0.15, 95% CI 0.00, 0.30, p = 0.05), number of sources likely to seek help from (n = 185, mean difference = 0.96, 95% CI 0.62, 1.30, p < 0.0001) and the level of mental health knowledge increased across the sample (n = 167, mean difference = 0.98, 95% CI 0.45, 1.50, p = 0.0003). There was no difference in self-help confidence, perceived peer support or mental well-being from baseline to follow-up, although baseline scores were already high: 34.3 (out of 48) and 30.0 (out of 40) for self help confidence and peer support, respectively.

**Table 5:** Paired sample t-tests comparing baseline and follow-up scores for help-seeking intentions, help-seeking confidence, mental health knowledge, perceived peer support and mental well-being for those with data at both time points

	Baseline	Follow-up	Within-group difference (95% CI)	*p*-value
	Mean (SD)	Mean (SD)		
Help-seeking intentions				
*n* = 185	3.22 (1.0)	3.37 (1.05)	0.15 (0.00023, 0.30)	0.05
Number of sources likely to seek help from				
*n* = 185	2.19 (1.85)	3.15 (2.33)	0.96 (0.62, 1.30)	** < 0.0001**
Self-help confidence				
*n* = 175	34.3 (4.94)	34.7 (4.68)	0.39 (− 0.28, 1.05)	0.25
Mental health knowledge				
*n* = 167	39.0 (3.92)	40.0 (3.84)	0.98 (0.45, 1.50)	**0.0003**
Peer support				
*n* = 159	30.0 (5.66)	30.4 (5.59)	0.33 (− 0.44, 1.10)	0.38
Mental well-being				
*n* = 159	22.3 (5.59)	22.3 (5.92)	0.06 (− 0.61, 0.72)	0.87

When conducting exploratory analysis of the effect of gender, student type, target peer educator year group, and peer learner year group in regression models, there was a larger mean change in wellbeing for males versus females (β = 1.81, 95% CI 0.28, 3.33, p = 0.020; Table 6) and in students who did not identify as either male nor female (β = -2.57, -5.12, -0.012, p = 0.049). The results suggested the intervention promoted a larger benefit in mental wellbeing in males than females, whereas those who did not identify as male or female showed a lesser benefit than females. The mean wellbeing scores for males were higher than females at baseline (24.5 vs 21.9), and at follow-up (25.8 vs 22.0), suggesting that PEP may actually widen gender inequalities. However, the small sample size for the those who did not identify as male or female (n = 28) and the fact that the study was not designed to be powered for these analyses meant that the confidence intervals were wide (see Table 2 for the sample sizes of the gender categories), and before accounting for school, there was no effect of this gender subgroup, while the other estimates remained the same (see Table S7). There was no evidence for an effect of the other three independent variables (student type, target peer educator year group, target peer learner year group) on any of the outcomes assessed.

**Table 6:** Regression results to examine the effect of gender, student type, target peer educator and peer learner year group on mean scores at follow-up, adjusted for baseline values and school ID

	Help-seeking intentions		Number of help sources likely to seek help from*		Help-seeking confidence		Mental health knowledge		Peer support		Mental well-being	
	β (95% CI)	*p*-value	β (95% CI)	*p*-value	β (95% CI)	*p*-value	β (95% CI)	*p*-value	β (95% CI)	*p*-value	β (95% CI)	*p*-value
Gender (female = ref)												
Male	− 0.018 (− 0.36, 0.32)	0.92	− 0.40 (− 1.21, 0.41)	0.33	0.62 (− 0.87, 2.10)	0.41	0.31 (− 0.84, 1.46)	0.60	− 0.12 (− 1.79, 1.55)	0.89	1.81 (0.28, 3.33)	**0.020**
Other	− 0.13 (− 0.66, 0.40)	0.64	− 0.45 (− 1.72, 0.81)	0.48	− 0.69 (− 3.02, 1.64)	0.56	1.26 (− 0.65, 3.17)	0.20	1.41 (− 1.40, 4.21)	0.32	− 2.57 (− 5.12, − 0.01)	**0.049**
Student type (peer learner = ref)												
Peer educator	0.0045 (− 0.31, 0.32)	0.98	0.094 (− 0.64, 0.83)	0.80	0.22 (− 1.13, 1.56)	0.75	0.074 (− 1.07, 1.21)	0.90	0.23 (− 1.44, 1.89)	0.79	− 0.94 (− 2.42, 0.55)	0.22
Target peer educator age (non-year 12 = ref)												
Year 12	− 0.18 (− 0.58, 0.22)	0.39	− 0.48 (− 1.44, 0.47)	0.32	− 0.085 (− 1.79, 1.62)	0.92	0.28 (− 1.11, 1.66)	0.69	− 0.83 (− 2.97, 1.31)	0.44	0.69 (− 1.20, 2.59)	0.47
Target peer learner age (year 7 = ref)												
Non− year 7	0.25 (− 0.10, 0.60)	0.16	0.67 (− 0.16, 1.49)	0.11	− 0.14 (− 1.66, 0.39)	0.86	− 0.38 (− 1.58, 0.83)	0.54	− 0.34 (− 2.13, 1.44)	0.71	− 1.24 (− 2.87, 0.39)	0.14

### Sample Size for a Powered Trial

We calculated the estimated sample size needed based on varied intra-cluster correlation coefficients (Table S8) and for three potential future primary outcomes, selected because we hypothesised they would be the most proximal points of change following the intervention (help-seeking intentions, self-help confidence and mental health knowledge), shown in Table S9. Sample size estimations varied, from seven to 15 schools in each arm with 120 students per school (for a primary outcome of mental health knowledge); to 13–18 schools in each arm for a primary outcome of self-help confidence, and 16–26 schools in each arm using a primary outcome of help-seeking intentions (the intra-cluster coefficient for this scale was very high).

## Discussion

We assessed the feasibility of undertaking a larger-scale evaluation of the PEP in secondary schools. Regarding attrition and completion of measures, all schools delivered the PEP, however one of the six schools did not complete any follow-up measures. Baseline rates of survey completion were high in three schools (from 69 to 79%), however for the other three schools fewer than 40% of eligible students completed the baseline measure. Eligibility was inferred from the number of unique email addresses provided by each school to send surveys to; overall, 42% of eligible students completed the baseline measures. Follow-up rates varied, ranging from a maximum of 69% to only 7% for one school. This suggests that the mode of delivery for the measures (online and unsupervised by researchers), would not be suitable for a future trial. Schools and students in the current study completed their survey electronically through a personal email link, and we conclude from the poor rates that schools either need to set aside lesson time and monitor students’ completion of measures, or researchers should physically visit schools to collect baseline and follow-up data (Kidger et al., 2021). We did however recruit and retain a varied sample of schools, which is a strength of this study. The feasibility of the data collection technique was also impacted by school-level characteristics. The school that did not complete follow-up data collection did not have a member of the senior leadership team who was accessible and appeared engaged with the study, and the IT team had data security concerns over sharing school email addresses (in spite of ethical approval and GDPR-compliant procedures on the part of the research team). Senior staff buy-in and support for similar research, as well as clear communications with IT staff around the sharing of such data need to be carefully considered in future work.

We found the reliability of the questionnaires to be satisfactory for the new measures, and excellent for the established measures. The mental health knowledge scale had a lower reliability overall and a lower test–retest reliability estimate than the other measures, suggesting further work may need to be conducted to refine and establish stronger psychometric properties for this newly-developed measure. The intra-cluster correlation coefficient for this measure was also very high, suggesting large variation between schools in mental health knowledge at baseline. This may indicate differences in the ways in which schools are highlighting mental health knowledge prior to the implementation of PEP, and this may be an important factor to measure in a future evaluation. EFA of the self help confidence items included one factor relating to self-management and communication, whereas the second factor included items pertaining to finding information. Further evaluation could validate these constructs and explore removing any redundant items to reduce the participant burden involved in completing multiple measures.

We found indications of change in the expected direction from baseline to follow-up for three of the outcome measures; help-seeking intentions, number of sources likely to seek help from, and mental health knowledge. This shows that the PEP was promising in terms of achieving proximal impacts on intentions to seek help and knowledge about mental health. However, confidence in self help, peer support and mental wellbeing did not indicate significant improvement from baseline to follow-up. Peer support and mental wellbeing changes may be more distal or expected to change in the longer-term post-intervention and therefore not sensitive to change in the short term. Interestingly, regression analyses showed that male students benefitted most from the intervention in terms of reporting a larger increase in wellbeing than other students. Given that males are less likely to seek help for mental health, this is in one way an encouraging finding that the PEP may lead to improved wellbeing in this population (Schonert-Reichl and Muller 1996), however as baseline measures of wellbeing were lower for girls than boys, the other impact of this finding is that PEP may actually increase gender inequalities in wellbeing further. A larger sample size and longer-term follow-up will be required to assess whether these changes can be replicated and are sustained following PEP, and to further explore differential effects by gender over time. This analysis was limited by the small proportion of participants who completed both the baseline and follow-up survey, so power to detect differences was limited. In addition, the length of time from post-intervention to survey completion varied from one to 12 weeks. The time elapsed may well play a role in the direction or magnitude of effects, however given the small sample of schools and not having measures of within and between-school variation (in terms of the final date of lesson delivery for each student and individual student survey response date), we were not able to investigate this. A future study should ideally capture these data so that the follow-up time in relation to changes in outcomes can be robustly assessed.

Many of the schools did not deliver the intervention as specified in terms of year group (delivered by year 12 to year 7); however this did not impact the associations with changes in the outcome, suggesting that the target age groups are not necessarily a key component of future effective PEP delivery. The MHF have designed the PEP with materials tailored to early adolescence soon after the transition from primary school, however several schools chose to deliver the PEP to year 8 students rather than year 7 (with the two that delivered to year 7 being single-sex fee-paying schools; the distinct characteristics of these schools may have confounded this finding). We are unsure whether this is due to the ongoing pandemic and schools feeling that their current year 8s had missed out on the opportunity of PEP the previous year; further research should investigate schools’ decision-making process in choosing the year groups to be peer learners.

Given the above findings, self-help confidence, help-seeking intentions or mental health knowledge may be appropriate primary outcomes for a future effectiveness trial of PEP, although the questions measuring mental health knowledge did not have very high reliability estimates and may need further refining should this measure be considered for a primary outcome. Self help confidence as a primary outcome would require a sample of around 18 schools in each arm, each with 120 students retained over the course of the study to have power of 90% in detecting an effect size of 0.30, and help-seeking intentions as primary outcome would require a sample size of 7 schools in each arm to achieve the same power. This size of effect would represent a small change overall in terms of the measures used, increasing scores on one or two questions overall. Incorporating these quantitative findings alongside the ongoing qualitative analysis may allow further insights into whether PEP is an appropriate and potentially effective means to increase mental health knowledge and help-seeking in the UK teen population. We found schools tended to remain in the study, but the survey measures were poorly completed, which was of interest and is a key learning point for the design of a future evaluation. However, it should be noted that the entire study was delivered during a pandemic, when mitigation measures regarding isolation in cases of Covid-19 were still in place and severely disrupting school attendance.

### Strengths and Limitations

This study had several strengths, recruiting six intervention schools and retaining five across two challenging years for schools and students (2021 and 2022). We assessed a broad range of indicators as to the feasibility of a future quantitative evaluation of the intervention and are able to draw key learning points from these findings, including refining measures and further assessing psychometric properties of the proposed new measures, ascertaining that established measures with hypothesised proximal effects appear sensitive to change following the intervention, and establishing estimates of the sample size needed to assess effectiveness in a cluster randomised controlled trial. There were some key limitations to the study however: firstly, there were marked differences between the intervention schools and the validation school, which should be taken into account when assessing test–retest reliability against the other psychometric properties of the measures. The validation school was more ethnically diverse than the intervention schools, which does increase the potential generalisability of the test–retest findings. There were different baseline scores in the measures between the intervention and validation schools that we consider may be attributable to calendar time: the intervention schools completed baseline measures in 2020 or early 2021 (during periods of national lockdowns and school closures), whereas the validation school completed their questionnaires in December 2021 (when lockdowns had been over for some months). In addition, one school had run the PEP for the preceding 3 years, which meant their baseline scores may have been higher to start with if the PEP sits within a wider framework of mental health provision within the school. Therefore, differences in the magnitude of change between new and existing user schools could be explored as an additional effect modifier in a future trial. We did not measure adherence to the intervention or record the dose (number of sessions delivered) or reach (number of students involved) in the feasibility study, this would need exploring in a future trial. There were also some avoidable errors in the mode of data collection that meant participants were able to give multiple responses to some questions. This only affected 10–15 students for most questions, however we had to treat these data as missing as we were not able to ascertain which response was the most accurate. This could be avoided through using different survey settings should this mode of data collection be repeated, however our other findings imply that researcher-supported data collection is essential for high response rates in a future trial. In conclusion, PEP is a feasible intervention and the design of our evaluation would be suitable for examining effectiveness in a fully powered trial, provided that sufficient sample size can be achieved and data collection is actively supported by a research team. Results from the realist evaluation, that examines the theory of change for this intervention, will be published shortly.

## Supplementary Information

Below is the link to the electronic supplementary material.

Supplementary file1 (DOCX 117 kb)

## References

[CR1] Aebi M, Giger J, Plattner B, Metzke CW, Steinhausen HC (2014). Problem coping skills, psychosocial adversities and mental health problems in children and adolescents as predictors of criminal outcomes in young adulthood. European Child and Adolescent Psychiatry..

[CR2] Asselmann E, Wittchen HU, Lieb R, Beesdo-Baum K (2018). Sociodemographic, clinical, and functional long-term outcomes in adolescents and young adults with mental disorders. Acta Psychiatrica Scandinavica..

[CR3] Bandura A, Walters RH (1977). Social learning theory.

[CR4] Boyce W, Torsheim T, Currie C, Zambon A (2006). The family affluence scale as a measure of national wealth: Validation of an adolescent self-report measure. Social Indicators Research.

[CR5] Children’s Commissioner for England, Briefing on Children’s Mental Health Services – 2020/2021. Retrieved April 28, 2022, from https://www.childrenscommissioner.gov.uk/report/briefing-on-childrens-mental-health-services-2020-2021/

[CR6] Colizzi, M., Lasalvia, A., Ruggeri, M. (2020) Prevention and early intervention in youth mental health: is it time for a multidisciplinary and trans-diagnostic model for care*? International Journal of Mental Health Systems* 14:1. 2020 Mar 24;14(1):1–14. 10.1186/s13033-020-00356-9 10.1186/s13033-020-00356-9 PMC7092613 32226481

[CR7] Copeland WE, Wolke D, Shanahan L, Costello J (2015). Adult functional outcomes of common childhood psychiatric problems: A prospective, longitudinal study. JAMA Psychiatry.

[CR8] Eldridge SM, Lancaster GA, Campbell MJ, Thabane L, Hopewell S, Coleman CL (2016). Defining feasibility and pilot studies in preparation for randomised controlled trials: Development of a conceptual framework. PLoS ONE.

[CR9] Harden A, Oakley A, Oliver S (2016). Peer-delivered health promotion for young people: A systematic review of different study designs. Health Education Journal.

[CR10] Harden, A., Weston, R., & Oakley, A. (1999). A review of the effectiveness and appropriateness of peer-delivered health promotion interventions for young people. *Database of abstracts of reviews of effects (DARE): Quality-assessed reviews*.

[CR11] Kidger J, Heron J, Lewis G, Evans J, Gunnell D (2012). Adolescent self-harm and suicidal thoughts in the ALSPAC cohort: A self-report survey in England. BMC Psychiatry.

[CR12] Kidger J, Turner N, Hollingworth W, Evans R, Bell S, Brockman R, Copeland L, Fisher H, Harding S, Powell J, Araya R, Campbell R, Ford T, Gunnell D, Morris R (2021). An intervention to improve teacher well-being support and training to support students in UK high schools (the WISE study): A cluster randomised controlled trial. PLoS Medicine.

[CR13] King T, Fazel M (2021). Examining the mental health outcomes of school-based peer-led interventions on young people: A scoping review of range and a systematic review of effectiveness. PLoS ONE.

[CR14] Langley AK, Nadeem E, Kataoka SH, Stein BD, Jaycox LH (2010). Evidence-based mental health programs in schools: Barriers and facilitators of successful implementation. School Mental Health.

[CR15] Mason H (2003). The facts peer education: Promoting healthy behaviors.

[CR16] NHS Digital. Mental Health of Children and Young People in England 2021—wave 2 follow up to the 2017 (2021). Accessed 12 July, 2022, from https://digital.nhs.uk/data-and-information/publications/statistical/mental-health-of-children-and-young-people-in-england/2021-follow-up-to-the-2017-survey2.

[CR17] Olivari C, Guzmán-González M (2017). Validation of the general help-seeking questionnaire for mental health problems in adolescents. Revista Chilena De Pediatria.

[CR18] O’Reilly M, Svirydzenka N, Adams S, Dogra N (2018). Review of mental health promotion interventions in schools. Social Psychiatry and Psychiatric Epidemiology.

[CR19] Parker K, Nunns M, Xiao ZM, Ford T, Ukoumunne OC (2021). Characteristics and practices of school-based cluster randomised controlled trials for improving health outcomes in pupils in the United Kingdom: a methodological systematic review. BMC Medical Research Methodology.

[CR20] Patalay P, Fitzsimons E (2021). Psychological distress, self-harm and attempted suicide in UK 17-year olds: Prevalence and sociodemographic inequalities. The British Journal of Psychiatry.

[CR21] Polissar L, Diehr P (1982). Regression analysis in health services research: the use of dummy variables. Medical Care.

[CR22] Sallis H, Szekely E, Neumann A, Jolicoeur-Martineau A, van Ijzendoorn M, Hillegers M (2019). General psychopathology, internalising and externalising in children and functional outcomes in late adolescence. Journal of Child Psychology and Psychiatry.

[CR23] Shiner M (1999). Defining peer education. Journal of Adolescence..

[CR24] Stewart-Brown S, Tennant A, Tennant R, Platt S, Parkinson J, Weich S (2009). Internal construct validity of the Warwick-Edinburgh Mental Well-Being Scale (WEMWBS): A Rasch analysis using data from the Scottish Health Education Population Survey. Health and Quality of Life Outcomes..

[CR25] Weare K (2013). Promoting mental, emotional and social health: A whole school approach.

[CR26] Wilson CJ, Deane FP, Ciarrochi JV, Rickwood D (2005). Measuring help seeking intentions: Properties of the general help seeking questionnaire. Canadian Journal of Counselling.

[CR27] Xia Y, Yang Y (2018). RMSEA, CFI, and TLI in structural equation modeling with ordered categorical data: The story they tell depends on the estimation methods. Behavior Research Methods..

