# Supplementary material for: Assessing the Feasibility of a Peer Education Project to Improve Mental Health Literacy in Adolescents in the UK
Source: Community Ment Health J. 2023 Jan 16;59(4):784–96. doi: 10.1007/s10597-022-01059-w (PMC9841483; doi:10.1007/s10597-022-01059-w)
Supplement: Supplementary file 1 — Supplementary file1 (DOCX 117 kb) [file 10597_2022_1059_MOESM1_ESM.docx]

## Figure S1. Flow diagram of participants through the study. Unit of clusters = school.


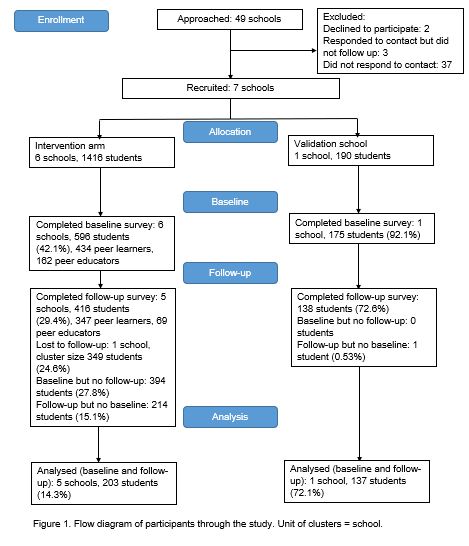


## Table S1. Descriptive statistics of study variables at baseline for those with complete baseline and follow-up data and those lost to follow-up.

|  | **Pre and post scores** | **Pre not post scores** | **p-value** |
| --- | --- | --- | --- |
| **Gender, % female** | n = 203 | n = 394 | **< 0.0001** |
|  | 58.1% | 54.1% |  |
| **School year, % year 7** | n = 203 | n = 387 | **< 0.0001** |
|  | 37.4% | 42.3% |  |
| **FAS, mean (SD)** | n = 183 | n = 325 | 0.15 |
|  | 9.19 (2.27) | 9.49 (2.24) |  |
| **Student status, % peer learner** | n = 203 | n = 384 | 0.070 |
|  | 68.0% | 75.0% |  |
| **Mean help-seeking intentions, mean (SD)** | n = 198 | n = 381 | **0.0006** |
|  | 3.21 (0.96) | 3.52 (1.09) |  |
| **Number of help sources, mean (SD)** | n = 198 | n = 381 | **0.0003** |
|  | 2.14 (1.82) | 2.79 (2.19) |  |
| **Self help confidence, mean (SD)** | n = 192 | n = 366 | **0.045** |
|  | 34.2 (5.00) | 35.1 (4.98) |  |
| **Mental health knowledge, mean (SD)** | n = 188 | n = 346 | 0.73 |
|  | 39.0 (3.84) | 39.2 (3.63) |  |
| **Perceived peer support, mean (SD)** | n = 186 | n = 336 | 0.13 |
|  | 30.1 (5.52) | 29.3 (6.54) |  |
| **Mental well-being, mean (SD)** | n = 186 | n = 333 | 0.63 |
|  | 22.1 (5.47) | 22.4 (5.30) |  |

## Treatment of missing data

In the ‘original dataset’, we coded responses to an individual question as missing if the participant had checked multiple answers, or if they had not responded. We then used a sensitivity approach to generate an alternate ‘imputed’ dataset maximising the data and sample size for analyses of change from baseline to follow-up (Eldridge et al., 2016). For those with ≤2 missing responses on questionnaires with 11 items or fewer, or for those with ≤3 missing values on scales with 12 or more total items, we replaced missing values with the individual’s mean response value on that scale (as recommended for the GHSQ and WEMWBS)(Olivari et al., 2017; Stewart-Brown et al., 2009). Participants with more items missing than this on a single questionnaire were excluded from the analysis of that measure. Due to a flaw in the online survey system, some participants gave more than one response to some questions. After eyeballing the data and seeing no discernable patterns in multiple responses, we decided to code these questions as missing and mean scores were imputed as above. Using t-tests and chi^2^ tests, we compared those with data at baseline and follow-up with those who only had baseline (lost to follow-up).

## Methods information for factor analysis

Confirmatory factor analysis (CFA) was also conducted using structural equation modelling commands and full information maximum likelihood estimation in Stata v17 for the help-seeking confidence scale to assess whether the following hypothesised groupings found in existing literature were evidenced: i) confidence to talk about own mental health (3 items) ii) confidence to find information/sources to deal with own mental health (6 items) and iii) confidence to deal with friend’s mental health (3 items), this was contrasted with the optimal solution suggested by the EFA. Finally, two CFA models were applied to assess whether help-seeking intention scores clustered into the following 3 or 4 groups (after consulting the literature and the mental health foundation on what they deemed as plausible, going from the most proximal to the most distal potential impacts of the intervention): 1. Friend, older student in my school (student-based) 2. Teacher, older adult in my school (school-based) 3. Parent, other relative, adult outside of my school, mental health professional, doctor, phone, online (outside school); Further decomposed for the four-factor model as: 3. Parent, other relative, adult outside of my school (outside school informal); 4. Mental health professional, doctor, phone, online (outside school formal). Model fit statistics, including the root mean square error of approximation (RMSEA), Tucker-Lewis index (TLI) and comparative fit index (CFI) were calculated and reported. Based on the simulation study of continuous data conducted by Hu and Bentler (1999), a good model fit was anticipated to be reflected by CFI and TLI of >0.95, and an RMSEA of 0.06 or smaller. A likelihood ratio test was used to assess whether the 3-factor or 4-factor model of help-seeking intentions was a better fit.

## Table S2. Reliability statistics for all measures at baseline (Cronbach’s alpha)

| **Scale** | **Cronbach's alpha** | | | | | |
| --- | --- | --- | --- | --- | --- | --- |
|  | **All at baseline** | | **Peer learners** | | **Peer educators** | |
|  | **Inter-item covariance average** | **Alpha coefficient** | **Inter-item covariance average** | **Alpha coefficient** | **Inter-item covariance average** | **Alpha coefficient** |
| General help seeking (all) 11 items | 0.87 | 0.84 | 0.92 | 0.85 | 0.61 | 0.80 |
| General help seeking student-based: 2 items | 0.47 | 0.36 | 0.50 | 0.37 | 0.47 | 0.40 |
| General help seeking school-based: 2 items | 1.86 | 0.85 | 2.04 | 0.87 | 0.99 | 0.69 |
| General help seeking outside school informal: 3 items | 1.21 | 0.69 | 1.13 | 0.68 | 1.04 | 0.64 |
| General help seeking outside school formal: 4 items | 1.45 | 0.78 | 1.57 | 0.80 | 0.95 | 0.72 |
| General help seeking within school sources: 4 items | 0.84 | 0.69 | 0.91 | 0.71 | 0.67 | 0.69 |
| General help seeking outside of school sources: 7 items | 1.03 | 0.79 | 1.10 | 0.80 | 0.63 | 0.70 |
|  |  |  |  |  |  |  |
| Confidence statements (all) 12 items | 0.13 | 0.78 | 0.15 | 0.80 | 0.09 | 0.72 |
| Confidence statements talking: 3 items | 0.21 | 0.63 | 0.22 | 0.65 | 0.19 | 0.62 |
| Confidence statements information: 3 items | 0.30 | 0.77 | 0.30 | 0.76 | 0.33 | 0.86 |
| Confidence statements coping: 3 items | 0.30 | 0.69 | 0.32 | 0.70 | 0.23 | 0.65 |
| Confidence statements friends: 3 items | 0.09 | 0.41 | 0.10 | 0.43 | 0.06 | 0.38 |
|  |  |  |  |  |  |  |
| Mental health knowledge (all) 12 items | 0.05 | 0.59 | 0.04 | 0.55 | 0.05 | 0.65 |
| Mental health knowledge 10 items removing two with low correlations | 0.06 | 0.60 | 0.05 | 0.54 | 0.07 | 0.68 |
|  |  |  |  |  |  |  |
| Sense of belonging peer relationships (all) 8 items | 0.45 | 0.80 | 0.45 | 0.78 | 0.31 | 0.80 |
| SWEBWBS (all) 7 items | 0.48 | 0.85 | 0.49 | 0.84 | 0.39 | 0.84 |

SWEMWBS: Short Warwick-Edinburgh Mental Wellbeing Scale

## Table S3. Intraclass correlation coefficients and confidence intervals, test-retest reliability over one month

|  | Main dataset | | | | Imputed dataset | | | |
| --- | --- | --- | --- | --- | --- | --- | --- | --- |
|  | Coefficient | 95% CI (lower) | 95% CI (upper) | p | Coefficient | 95% CI (lower) | 95% CI (upper) | p |
| General help seeking | 0.69 | 0.69 | 0.82 | <0.001 | 0.71 | 0.69 | 0.82 | <0.001 |
| Confidence statements | 0.67 | 0.66 | 0.80 | <0.001 | 0.64 | 0.60 | 0.76 | <0.001 |
| Mental health knowledge | 0.50 | 0.47 | 0.68 | <0.001 | 0.50 | 0.45 | 0.66 | <0.001 |
| Sense of belonging: peer relationships | 0.79 | 0.76 | 0.86 | <0.001 | 0.79 | 0.76 | 0.86 | <0.001 |
| SWEMWBS | 0.73 | 0.69 | 0.82 | <0.001 | 0.71 | 0.66 | 0.80 | <0.001 |

SWEMWBS: Short Warwick-Edinburgh Mental Wellbeing Scale

## Table S4. Factor loadings for exploratory factor analysis of help-seeking confidence questionnaire

| Variable | Factor1 | Factor2 | Factor3 | Uniqueness |
| --- | --- | --- | --- | --- |
|  |  |  |  |  |
| con_friends1 | 0.3038 | 0.0768 | 0.116 | 0.8538 |
| con_friends2 | -0.0895 | 0.0801 | 0.5473 | 0.7044 |
| con_talk_q1 | 0.5775 | -0.0218 | 0.1039 | 0.6427 |
| con_talk_q2 | 0.7317 | 0.0204 | -0.0064 | 0.4536 |
| con_talk_q3 | 0.4513 | -0.1349 | 0.0461 | 0.8196 |
| con_info_q1 | 0.0139 | 0.7422 | -0.0349 | 0.4433 |
| con_info_q2 | 0.2079 | 0.5168 | 0.0582 | 0.5875 |
| con_info_q3 | -0.0317 | 0.8834 | 0.0102 | 0.2406 |
| con_cope_q1 | 0.6987 | 0.0143 | -0.079 | 0.5188 |
| con_cope_q2 | 0.7857 | 0.0138 | -0.0019 | 0.374 |
| con_cope_q3 | 0.4198 | 0.0736 | 0.0741 | 0.7741 |
| con_frien~3r | 0.0395 | -0.0366 | 0.7035 | 0.4965 |

| **Factor 1** | **Factor 2** | **Factor 3** |
| --- | --- | --- |
| would tell a friend to speak to an adult if worried about their mental health | knowing where to get information in school to look after my mental health | if I was worried about a friends mental health I; would talk to a friend about their mental health |
| feel OK talking about my mental health with other people | knowing who I can talk to if I want to know more about mental health | if I was worried about a friends mental health I would [not] be too embarrassed to do anything about it |
| Knowing how to explain to someone about how I’m feeling | knowing where to get help and support for my mental health in school |  |
| talking to someone about mental health in spite of how they might react |  |  |
| Knowing how to take care of my mental health |  |  |
| Knowing when to ask for help with how I am feeling |  |  |
| Knowing how to use a breathing exercise to manage how I am feeling |  |  |

## Table S5. Factor loadings for exploratory factor analysis of mental health knowledge

| Variable | Factor1 | Factor2 | Uniqueness |
| --- | --- | --- | --- |
| truefalse_1 | 0.2709 | 0.1411 | 0.8802 |
| truefalse_2r | 0.0513 | 0.3602 | 0.8548 |
| truefalse_3 | 0.2968 | 0.0078 | 0.9102 |
| truefalse_4r | 0.0987 | 0.3265 | 0.8613 |
| truefalse_5 | 0.5157 | -0.2161 | 0.7648 |
| truefalse_6r | 0.2999 | -0.0757 | 0.9201 |
| truefalse_7 | -0.1061 | 0.483 | 0.7911 |
| truefalse_8 | 0.1301 | 0.5157 | 0.6706 |
| truefalse_9 | 0.0003 | 0.1593 | 0.9746 |
| truefalse_10 | 0.0194 | 0.4271 | 0.8114 |
| truefalse_11 | 0.5356 | 0.1583 | 0.6292 |
| truefalse_12 | 0.4872 | 0.1327 | 0.7002 |

## Items in each factor

| **Factor 1** | **Factor 2** |
| --- | --- |
| mental health is something we all have | having good mental health means there are no problems in your life |
| people with mental health problems can get better | very few people experience mental health problems |
| physical activity (exercise) can improve mental health | problems with friends or classmates can make your mental health worse |
| there’s not much you can do to help a friend with a mental health problem | the environment people live and grow up in can affect our mental health |
| the amount of sleep people get can affect how they feel | noticing or paying attention to negative emotions can make them worse |
| what people eat and drink can affect their mental health | people who have mental health problems can find it difficult to do school work |

## Table S6. Factor loadings and R^2^ for confirmatory factor analysis models.

|  | Confidence statements 3-factor model (author suggested) | | |  |  |  | Confidence statements 3-factor model (determined by EFA) | | | | GHS 3-factor model | | |  | GHS 4-factor model | |  |
| --- | --- | --- | --- | --- | --- | --- | --- | --- | --- | --- | --- | --- | --- | --- | --- | --- | --- |
|  | Variable | Factor loading | R squared |  |  |  |  |  |  |  |  | Factor loading | R squared |  | Factor loading | R squared |  |
|  |  |  |  |  |  |  |  |  |  |  |  |  |  |  |  |  |  |
| Factor 1 | con_talk_q1 | 0.61 | 0.37 | Factor 1 | con_friends1 | 0.35 | 0.12 | Factor 1 | ghs_q1_score | 0.24 | 0.06 | 0.25 | 0.06 | Factor 1 | ghs_q1_score | 0.24 | 0.06 |
|  | con_talk_q2 | 0.79 | 0.62 |  | con_talk_q1 | 0.58 | 0.34 |  | ghs_q6_score | 0.91 | 0.83 | 0.90 | 0.81 |  | ghs_q6_score | 0.91 | 0.83 |
|  | con_talk_q3 | 0.42 | 0.17 |  | con_talk_q2 | 0.73 | 0.54 | Factor 2 | ghs_q4_score | 0.85 | 0.72 | 0.86 | 0.74 | Factor 2 | ghs_q4_score | 0.85 | 0.72 |
| Factor 2 | con_info_q1 | 0.46 | 0.21 |  | con_talk_q3 | 0.41 | 0.17 |  | ghs_q5_score | 0.86 | 0.74 | 0.85 | 0.72 |  | ghs_q5_score | 0.86 | 0.74 |
|  | con_info_q2 | 0.55 | 0.31 |  | con_cope_q1 | 0.68 | 0.47 | Factor 3 | ghs_q2_score | 0.48 | 0.23 | 0.67 | 0.45 | Factor 3 | ghs_q2_score | 0.48 | 0.23 |
|  | con_info_q3 | 0.50 | 0.25 |  | con_cope_q2 | 0.78 | 0.61 |  | ghs_q3_score | 0.55 | 0.30 | 0.75 | 0.56 |  | ghs_q3_score | 0.55 | 0.30 |
|  | con_cope_q1 | 0.69 | 0.47 |  | con_cope_q3 | 0.47 | 0.22 |  | ghs_q7_score | 0.56 | 0.32 | 0.58 | 0.34 |  | ghs_q7_score | 0.56 | 0.32 |
|  | con_cope_q2 | 0.76 | 0.58 | Factor 2 | con_info_q1 | 0.75 | 0.56 |  | ghs_q8_score | 0.66 | 0.43 | 0.69 | 0.48 | Factor 4 | ghs_q8_score | 0.66 | 0.43 |
|  | con_cope_q3 | 0.47 | 0.23 |  | con_info_q2 | 0.61 | 0.37 |  | ghs_q9_score | 0.68 | 0.47 | 0.74 | 0.54 |  | ghs_q9_score | 0.68 | 0.47 |
| Factor 3 | con_friends1 | 0.22 | 0.05 |  | con_info_q3 | 0.85 | 0.72 |  | ghs_q10_sc~e | 0.69 | 0.48 | 0.76 | 0.57 |  | ghs_q10_sc~e | 0.69 | 0.48 |
|  | con_friends2 | 0.50 | 0.25 | Factor 3 | con_friends2 | 0.38 | 0.15 |  | ghs_q11_sc~e | 0.55 | 0.30 | 0.58 | 0.34 |  | ghs_q11_sc~e | 0.55 | 0.30 |
|  | con_frien~3r | 0.74 | 0.54 |  | con_frien~3r | 1.00 | 1.00 |  |  |  |  |  |  |  |  |  |  |
|  |  |  |  |  |  |  |  |  |  |  |  |  |  |  |  |  |  |
|  | Overall |  | 0.96 |  |  |  |  |  |  |  | OVERALL |  | 0.99 |  |  |  | 0.99 |

## Table S7. Comparison of mean scores at follow-up according to gender, student type, and whether the intervention was delivered with the target year groups, adjusted for baseline values.

|  | **Help-seeking intentions** | | **Number of sources likely to seek help from*** | | **Help-seeking confidence** | | **Mental health knowledge** | | **Peer support** | | **Mental well-being** | |
| --- | --- | --- | --- | --- | --- | --- | --- | --- | --- | --- | --- | --- |
|  | B (95% CI) | p-value | B (95% CI) | p-value | B (95% CI) | p-value | B (95% CI) | p-value | B (95% CI) | p-value | B (95% CI) | p-value |
| **Gender (female = ref)** | | | | | | | | | | | | |
| Male | -0.056  (-0.37, 0.26) | 0.73 | -0.37  (-1.12, 0.37) | 0.31 | 0.46  (-0.88, 1.81) | 0.50 | 0.32  (-0.73, 1.38) | 0.54 | -0.28  (-1.83, 1.26) | 0.72 | 1.68 (0.28, 3.08) | **0.019** |
| Other | -0.19  (-0.72, 0.35) | 0.49 | -0.62  (-1.89, 0.64) | 0.33 | -0.73  (-3.02, 1.56) | 0.53 | 1.29  (-0.59, 3.16) | 0.18 | 1.53  (-1.22, 4.28) | 0.27 | -2.42  (-4.95, 0.11) | 0.061 |
| **Student type (peer learner = ref)** | | | | | | | | | | | | |
| Peer educator | -0.01 (-0.30, 0.27) | 0.93 | 0.067  (-0.61, 0.74) | 0.85 | 0.084  (-1.15, 1.32) | 0.89 | 0.065  (-1.0, 1.13) | 0.90 | 0.41  (-1.14, 1.96) | 0.60 | -0.87  (-2.25, 0.51) | 0.22 |
| **Target peer educator age (non-year 12 = ref)** | | | | | | | | | | | | |
| Year 12 | -0.090  (-0.40, 0.22) | 0.57 | -0.042  (-0.78, 0.69) | 0.91 | -0.40  (-1.70, 0.91) | 0.55 | 0.12  (-0.96, 1.20) | 0.83 | -0.85  (-2.53, 0.83) | 0.32 | 0.26  (-1.28, 1.79) | 0.74 |
| **Target peer learner age (year 7 = ref)** | | | | | | | | | | | | |
| Non-year 7 | 0.22  (-0.05, 0.48) | 0.11 | 0.47  (-0.16, 1.10) | 0.15 | 0.15  (-1.02, 1.31) | 0.80 | -0.34  (-1.30, 0.61) | 0.48 | -0.66  (-2.05, 0.74) | 0.36 | -1.04  (-2.35, 0.27) | 0.12 |

*Help-seeking intentions as indicated by the number of sources likely-extremely likely to seek help from.

## Table S8. Intra-cluster correlation coefficients for potential future primary outcome measures

| **Measure** | **Baseline ICC** | **Lower CI** | **Upper CI** |
| --- | --- | --- | --- |
|  |  |  |  |
| **Average help-seeking intentions (general help-seeking questionnaire)** | 0.089 | 0.000 | 0.189 |
| **Self help confidence** | 0.070 | 0.000 | 0.165 |
| **Mental health knowledge** | 0.019 | 0.000 | 0.056 |

## Table S9. Sample size calculations

|  | **Primary outcome self-help confidence** | | | **Primary outcome help-seeking intentions** | | | **Primary outcome mental health knowledge** | | |
| --- | --- | --- | --- | --- | --- | --- | --- | --- | --- |
|  |  | Schools (in one arm) | Total N per arm |  | Schools (in one arm) | Total N per arm |  | Schools (in one arm) | Total N per arm |
| **1. Using estimated ICC** | Estimation of sample size needed for GHSQ with change of 0.3 points, SD 1.06, sample size of 120 per school at follow-up, and ICC of 0.09 | 18 | 2160 | Estimation of sample size needed for help-seeking confidence with change of 1.5 points, SD 4.87, sample size of 120 per school at follow-up, and ICC of 0.07 | 26 | 3120 | Estimation of sample size needed for mental health knowledge with change of 1.15 points, SD 3.94, sample size of 120 per school at follow-up, and ICC of 0.02 | 7 | 840 |
| **2. Using ICC 0.05** | Estimation of sample size needed for GHSQ with change of 0.3 points, SD 1.06, sample size of 120 per school at follow-up, and ICC of 0.05 | 13 | 1560 | Estimation of sample size needed for help-seeking confidence with change of 1.5 points, SD 4.87, sample size of 120 per school at follow-up, and ICC of 0.05 | 16 | 1920 | Estimation of sample size needed for mental health knowledge with change of 1.15 points, SD 3.94, sample size of 120 per school at follow-up, and ICC of 0.05 | 15 | 1800 |
